# Erratum: Xylose Metabolism and the Effect of Oxidative Stress on Lipid and Carotenoid Production in *Rhodotorula toruloides*: Insights for Future Biorefinery

**DOI:** 10.3389/fbioe.2020.617204

**Published:** 2020-10-30

**Authors:** Frontiers Production Office

**Affiliations:** Frontiers Media SA, Lausanne, Switzerland

**Keywords:** microbial oil, carotenoids, *Rhodotorula toruloides*, Genome-scale modelling, xylose, biorefinery, absolute proteomics

Due to a production error, incorrect text was used for the Acknowledgments and the Funding.

A correction has been made to the section **Funding**:

“This project has received funding from the European Union's Horizon 2020 research and innovation program under grant agreement no. 668997 and the Estonian Research Council (grant PUT1488P). MP would additionally like to acknowledge Coordination for the Improvement of Higher Education Personnel (Capes), São Paulo Research Foundation (FAPESP, grant 2016/10636-8) and DORA Plus.”

A correction has been made to the section **Acknowledgments**:

“We thank the Proteomics Core Laboratory at University of Tartu for the proteome quantification. A preprint (Pinheiro et al., 2020) has been deposited at bioRxiv doi: 10.1101/2020.05.28.121012”.

The publisher apologizes for this mistake. The original article has been updated.
