# Supplementary material for: Xylose Metabolism and the Effect of Oxidative Stress on Lipid and Carotenoid Production in Rhodotorula toruloides: Insights for Future Biorefinery
Source: Front Bioeng Biotechnol. 2020 Aug 19;8:1008. doi: 10.3389/fbioe.2020.01008 (PMC7466555; doi:10.3389/fbioe.2020.01008)
Supplement: Supplementary file 2 [file Image_1.pdf]

Supplementary Material

1 Supplementary Figures

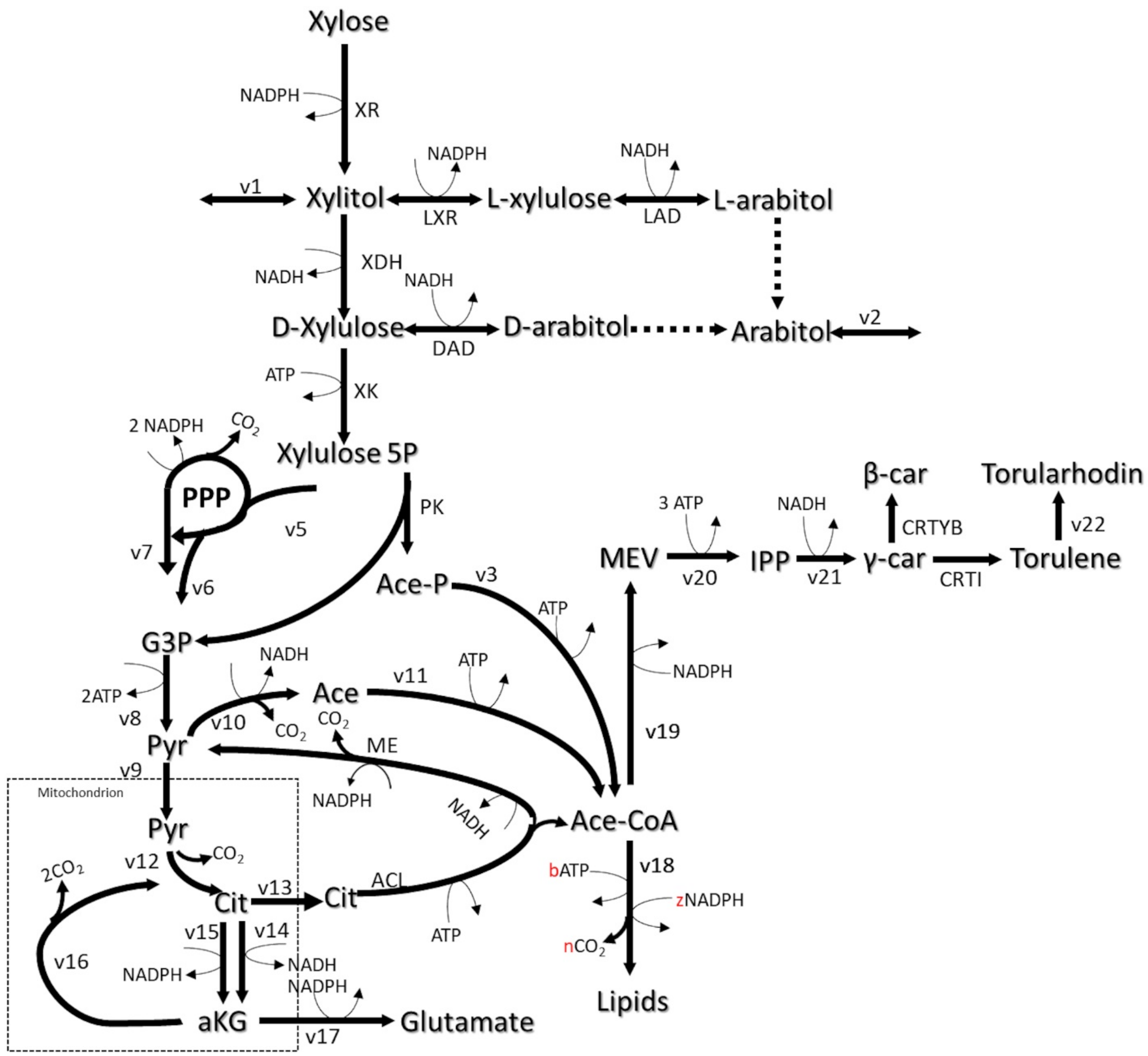

Figure S1. Schematic representation of the pathway leading to the consumption of xylose by *R. toruloides*. Dashed arrows comprise the *in silico* pseudo-reaction of D and L-arabitol into an "artificial" arabitol without a specific isoform. All fluxes and complete legend can be found in the Supplementary Table S4.

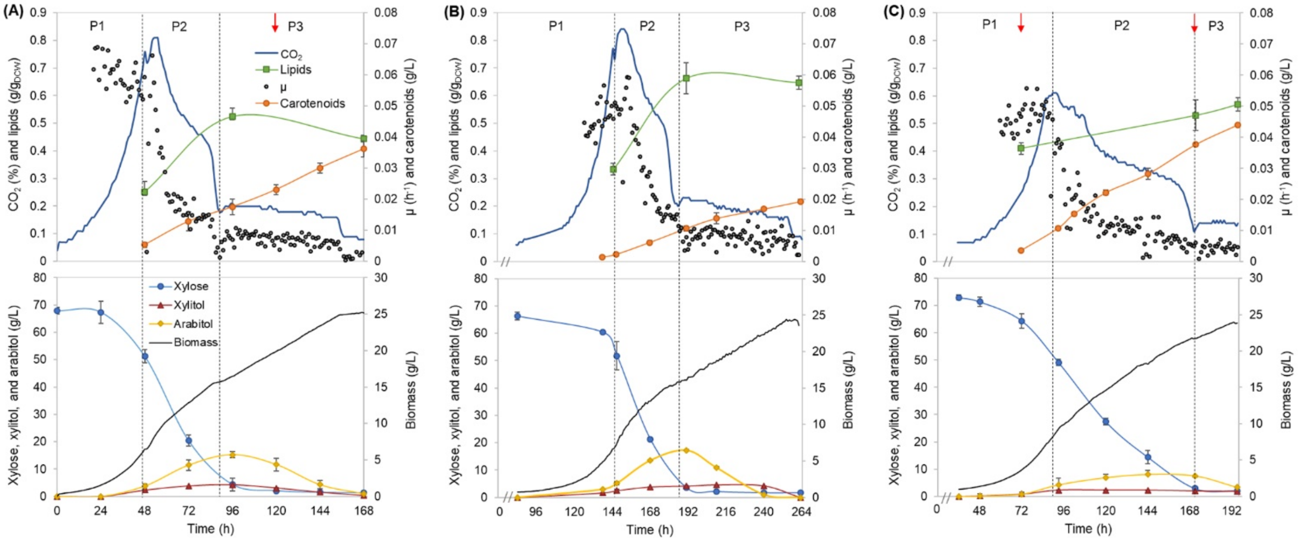

Figure S2. *R. toruloides* batch cultivation on xylose under light irradiation **(A)** and in presence of H_2O_2 **(B)**, and adapted strain under oxidative stress **(C)**. Dashed vertical lines define three observed growth phases. The specific growth rate (μ), biomass concentration, intracellularly accumulated lipid and carotenoid concentrations, extracellular metabolite profiles and CO_2 production profile in the outflow gas are presented. The values represent an average of three independent cultivation experiments; error bars represent standard deviation. The red arrows represent the proteomic data points.

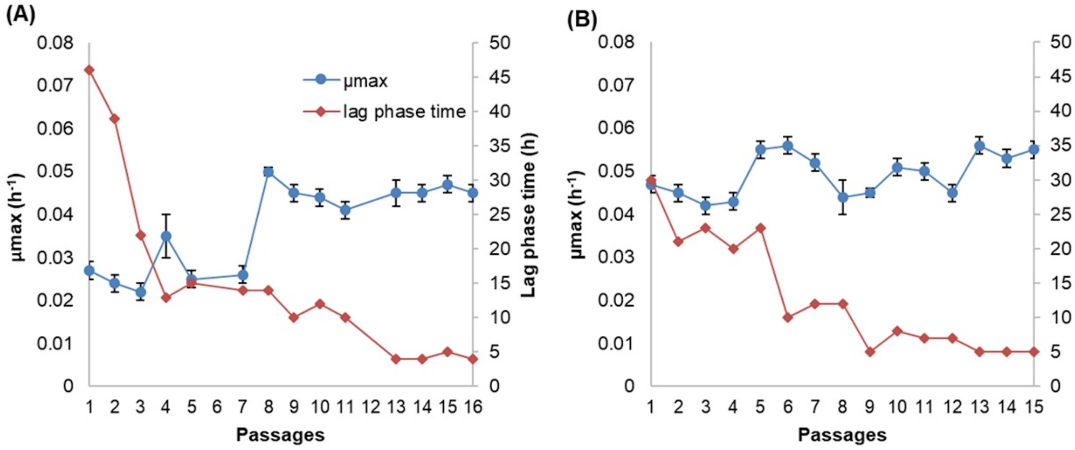

Figure S3. Changes in μ_{max} and lag phase time during the **(A)** 1st and **(B)** 2nd cycle of the adaptive laboratory evolution with H_2O_2. Error bars represent the standard deviation from three measurements.

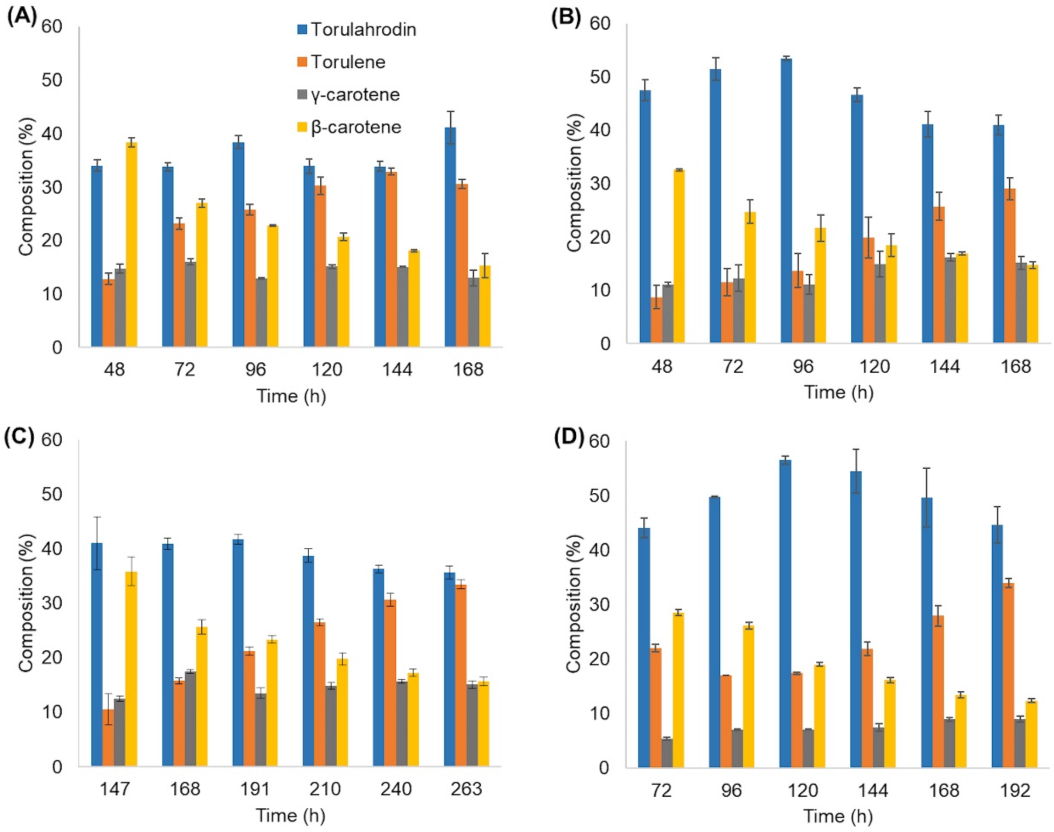

Figure S4. Carotenoids composition during the batch cultivation of *R. toruloides* under the studied conditions: reference condition (REF), under light irradiation (LIG) and in presence of hydrogen peroxide (PER), and the adapted strain under oxidative stress (ADA).
